# Management of Ludwig's Angina at a Tertiary Care Hospital in Western Region of India

**DOI:** 10.7759/cureus.23311

**Published:** 2022-03-19

**Authors:** Bhagirath D Parmar, Krupal J Joshi, Ankur D Modi, Gavendra P Dave, Raji S Desai

**Affiliations:** 1 Department of Otorhinolaryngology-Head and Neck Surgery, C U Shah Medical College and Hospital, Surendranagar, IND; 2 Department of Community and Family Medicine, All India Institute of Medical Sciences, Rajkot, IND; 3 Department of General Surgery, C U Shah Medical College and Hospital, Surendranagar, IND; 4 Department of Otorhinolaryngology-Head and Neck Surgery, Sir Takhatsinhji (T) Hospital and Government Medical College, Bhavnagar, IND

**Keywords:** incision and drainage, grodinsky criteria, tracheostomy, surgical decompression, submental and submaxillary space, floor of mouth cellulitis, dental caries

## Abstract

Introduction

Ludwig’s angina is cellulitis of submandibular space, submental space, and sublingual space. The main causative factors include dental infections (dental caries with atypical periodontitis, pericoronitis, and dental procedures). Other predisposing conditions include poor dental hygiene, dental caries, malnutrition, diabetes mellitus, AIDS, and various other immunocompromised states. It presents as an acute onset and spreads very rapidly causing bilateral diffuse neck swelling, edema of floor of mouth, pain, fever, trismus, foul-smelling pus discharge, difficulty in swallowing, airway edema, and tongue displacement creating a compromised airway with stridor. So it requires early diagnosis and aggressive management.

Material and methods

Clinical data of all patients with clinical diagnosis of Ludwig’s angina managed at the Department of Otorhinolaryngology-Head and Neck Surgery, Sir Takhatsinhji (T) General Hospital and Government Medical College, Bhavnagar, India, from 2015 to 2019 were analyzed retrospectively in this study.

Result

Over the review period, 30 cases were diagnosed as Ludwig’s angina, out of which 12 (40%) were males and 18 (60%) were females; male to female ratio was 1:1.5. The age of the patients ranged from six months to 64 years, with a mean age of 38.86 years. Fever, neck swelling, and neck pain were present in all patients.

In 16 patients, incision and drainage were done under general anesthesia while the rest five patients required only local anesthesia. In six patients (20%), for maintenance of airway, tracheostomy was required. The most common complication was necrotizing fasciitis and death followed by septicemia. Mortality was observed in three patients (10%) in this study.

Conclusion

Despite improved outcomes compare to pre-antibiotic era, Ludwig's angina still remains a potentially life-threatening disease in ENT at present. Dental caries, uncontrolled diabetes mellitus, and malnutrition are commonly associated conditions. With early diagnosis, close airway observation, aggressive intravenous antibiotic treatment, and timely surgical intervention, morbidity, and mortality can be reduced.

## Introduction

Angina is from a Greek word, "Anchone," meaning strangulation. Ludwig’s angina was first described by German physician Wilhelm Frederick Von Ludwig in 1836. Cellulitis is an infectious inflammation of the cellular adipose tissue located in the apo-neurotic spaces. Ludwig's angina is cellulitis of sub-mandibular space, sub-mental space, and sublingual space. Initially, swelling develops unilaterally and then spreads bilaterally. Edema and cellulitis of supra-omohyoid space may develop then after causing compression of airway and dysphagia [1]. Infection may spread to retro-pharyngeal space, para-pharyngeal space, and in some cases up to mediastinum resulting in difficulty in breathing. The possible ways of infection spread are destruction of facial planes, aspirations of infective particles, or septic embolism to pulmonary vasculature [2].

The incidence of Ludwig’s angina is nowadays less compared to pre-antibiotic era because of awareness regarding dental health and effective antibiotic therapy [3]. The origin is generally from the carious second and third molar. In fact, the roots of these teeth penetrate the mylohyoid ridge, such that any abscess or dental infection has direct access to the sub-maxillary space. The main causative factors include dental infections (dental caries with atypical periodontitis and pericoronitis). Other predisposing conditions include poor dental hygiene, malnutrition, other infections like sialadenitis, floor of mouth trauma, mandible fracture, diabetes mellitus, AIDS, and various immuno-compromised states [4]. The commonly underlying microorganisms are Streptococcus sp., *Staphylococcal aureus*, *Hemophilus influenza,* and some anaerobes like Bacteroids sp., Fusobacterium, etc. [5].

It presents as an acute onset and spreads very rapidly causing bilateral diffuse neck swelling, edema of the floor of mouth, pain, fever, trismus, difficulty in swallowing, airway edema, and tongue displacement causing compromised airway, stridor, foul-smelling pus discharge. So it requires early diagnosis and aggressive management. Diagnosis is mainly clinical and supported with radiological investigation to know the extent of spread and to discover dental origin. Plain x-ray neck is used to see the extent of soft tissue swelling, gas formation, and airway compression [6]. Ultrasonography is also helpful to access involvement of various spaces, size of collection, and also help in monitoring of prognosis. Culture and sensitivity of collected pus/fluid help in the choice of proper antibiotics. It is managed initially with empirical broad-spectrum intravenous antibiotics with anti-inflammatory drugs. Primary dental causes or associated factors are addressed simultaneously. In severe cases, surgical decompression along with maintenance of secured airway by tracheostomy may require. If not managed properly and aggressively, it can lead to fatal complications.

Grodinsky in 1939, proposed four criteria, to distinguish Ludwig's angina from other deep neck abscesses [5]. According to the criteria, the infection must (1) occur bilaterally in more than one space, (2) produce serosanguinous infiltration with or without pus, (3) involve connective tissue, fascia, and muscles but not glandular structures, and (4) spread by continuity, not by lymphatics.

## Materials and methods

Objectives

This study aimed to evaluate, find out, and compare various clinical features, underlying etiology and associated factors, management protocol, and complications in patients of Ludwig's angina who present within/after 72 hours (early/late presentation) to the hospital.

Study design

This study is a retrospective analysis of 30 cases of Ludwig’s angina managed in the Department of Otorhinolaryngology-Head and Neck Surgery, Sir Takhatsinhji (T) Hospital and Government Medical College, Bhavanagar, India, from 2015 to 2019. Collected data of patients like age, sex, socioeconomic status, predisposing condition, time interval of presentation, presenting signs and symptoms, radiological and pathological investigations, treatment protocol, complications, outcomes, and follow-up states were evaluated.

Inclusion and exclusion criteria

All the patients of age group between six months to 65 years irrespective of their gender were included in the study. Patients must fulfill Grodinsky’s criteria to satisfy the inclusion criteria of the study. The patients who did not admit and not fulfilled all four Grodinsky’s criteria were excluded from this study.

Statistical analysis

Data were collected, compiled, and analyzed using SPSS version 21.0 (Chicago, IL: IBM Corp.). For the analysis, we used measurements of central tendency like mean, standard deviation and median, confidence interval. For qualitative data analysis, we used chi-square test and Fisher's exact test to find out the association between two variables like complication, etiology, management protocol, early presentation, and late presentation.

## Results

Over the review period, 30 cases were diagnosed as Ludwig’s angina, out of which 12 (40%) were males and 18 (60%) were females; male to female ratio was 1:1.5. The age of the patients ranged from six months to 64 years, with a mean age of 38.86±7.80 years. Twenty-one (70%) patients were from lower socioeconomic class whereas seven (23.33%) patients were from middle and two (6.66%) from upper socioeconomic class. Out of 30 patients, nine were presented early to the hospital while the rest 21 patients presented late to the hospital. Duration of stay ranged from seven days to 60 days with a mean stay duration of 23 days.

Association of presenting complaints with early and late presentations is shown in Table 1. Fever, neck swelling, and neck pain were present in all patients. Dental infections and diabetes mellitus were the two most common underlying etiologies observed.

**Table 1:** Association of presenting symptoms with early and late presentations. Fisher's exact test was used to find out the association between symptoms with early and late presentations. The test value was 16.71 with a p-value of 0.05. Here, the p-value is less than 0.05, so the null hypothesis was rejected. It means there is an association of symptoms with early and late presentations (more than one symptom was present in all patients).

Symptoms	Number of patients	Percentage	Symptoms in early presentation	Symptoms in late presentation	Chi-square/Fisher's exact test	p-Value
Neck swelling	30	100%	9	21	16.71	0.05
Neck pain	30	100%	9	21		
Fever	30	100%	9	21		
Dysphagia	25	83.33%	4	21		
Dental pain	18	63.33%	12	6		
Trismus	13	43.33%	5	8		
Muffled voice	11	36.66%	0	11		
Fetid breath	8	26.66%	2	6		
Respiratory distress	6	20%	0	6		
Swelling in the floor of mouth	4	13.33%	2	2		

Association of etiology with early and late presentations is mentioned in Table 2. In 11 patients, caries of second and/or third molar root with exposed pulp was observed. In two patients, infection developed after tooth extraction. Three mentally retarded children (out of which two were male and one female) with poor oral hygiene developed infection. Two patients were admitted to medicine department for diabetic ketoacidosis and then were referred to ENT department for Ludwig’s angina. Details of bacterial organisms isolated in culture are shown in Table 3. In 24 patients, pus/serous fluid was sent for microbiological culture. The most common organism found was Streptococcus followed by *Escherichia coli*.

**Table 2:** Association of etiology with early and late presentations. Fisher's exact test was used to find out the association between etiology with early and late presentations. The test value was 7.095 with a p-value of 0.52. Here, the p-value is more than 0.05, so the null hypothesis was accepted. It means there is no association of etiology with early and late presentations (more than one underlying causative factor was present in one patient).

Etiologies	Present in number of patients	Percentage	Etiology in early presentation	Etiology in late presentation	Chi-square/Fisher's exact test	p-Value
Dental infection	19	63.33%	7	12	7.095	0.52
Diabetes mellitus	11	36.77	1	10		
Salivary-gland infection/sialoadenitis	8	26.66%	6	2		
Malnutrition	6	20%	1	5		
Oral laceration	3	10%	2	1		
Sharp foreign-body ingestion like fishbone	2	6.66%	0	2		
Chronic renal failure	2	6.66%	0	2		
Insect bite	1	3.33%	1	0		
Application and ingestion of some home remedies/herbal products	1	3.33%	1	0		

**Table 3:** Bacterial organisms isolated in culture.

Organisms	Number of patients	Percentage
Streptococcus	11	45.83%
Escherichia coli	5	20.83%
Staphylococcus	3	12.5%
Pseudomonas	1	4.16%
No growth	4	16.55%
Surgical-decompression	24	80%
Conservative medical management	6	20%

Association of management protocol with early and late presentations is illustrated in Table 4. In 21 cases, incision and drainage were done externally while in three cases, it was being done intra-oral route. In external route, incision was given from one angle of mandible to another for better drainage of serosanguinous fluid/pus with surgical decompression of gas. Frank pus was found in eight patients and serosanguinous fluid was found in 16 patients. In 16 patients, incision and drainage were done under general anesthesia while the rest five patients required only local anesthesia. In six patients (20%), for maintenance of airway, tracheostomy was required. A soft tissue neck x-ray was done in all patients except one pregnant female. In eight patients, contrast CT (computed tomography) scan neck was done to access the extent of infection spread. Ultrasonography was done in all cases for both diagnosis and prognosis.

**Table 4:** Association of management protocol with early and late presentations. Fisher's exact test was used to find out the association between management protocol with early and late presentations. The test value was 1.77 with a p-value of 0.62. Here, the p-value was more than 0.05, so the null hypothesis was accepted. It means there is no association of complication with early and late presentations.

Management protocol	In early presentation	In late presentation	Chi-square/Fisher's exact test	p-Value
Conservative medical management	02	04	1.77	0.62
Surgical-decompression (Incision and drainage)	07	11		
Surgical-decompression (Incision and drainage) + tracheostomy	00	06		

Association of complications with early and late presentations is shown in Table 5. The most common complication was necrotizing fasciitis (16.66%). Death (10%) and septicemia (10%) were the second common complications in this study. Mortality was observed in three patients (10%) in this study. Sudden death was observed 12 hours after surgical decompression in one diabetic middle-aged male patient who presented with diabetic ketoacidosis and the complications observed were septicemia followed by disseminated intravascular coagulation (DIC) and cardiac arrest. Another old-age female patient having some undiagnosed brain tumor died one month after drainage because of septicemic shock, DIC, and cardiac arrest. One younger pregnant female patient (with seven months amenorrhea) presented on the fourth day with a history of ingestion and application of some home remedies/herbal products and started deteriorating. In spite of aggressive intravenous antibiotics, tracheostomy, incision and drainage (I&D), and lower segment caesarian section, she ultimately died on the 11th day because of septicemia and mediastinitis. Delayed wound healing was observed in six patients (20%) because of uncontrolled diabetes mellitus, multiple dental infections, and poor nutritional status. In eight patients secondary suturing was required.

**Table 5:** Association of complications with early and late presentation. Fisher's exact test was used to find out the association between complications with early and late presentation. The test value was 1.51 with a p-value of 0.91. Here, the p-value was more than 0.05, the null hypothesis was accepted. It means there is no association of complication with early and late presentations.

Complications	Number of patients	Complications observed in early presentation	Complications observed in late presentation	Fisher's exact test	p-Value
Necrotizing fasciitis	5 (16.66%)	2	3	1.51	0.91
Death	3 (10%)	0	3		
Septicemia	3 (10%)	1	2		
Disseminated intravascular coagulation (DIC)	2 (6.67%)	0	2		
Mediastinitis	1 (3.33%)	0	1		
Cardiac arrest	1 (3.33%)	0	1		

## Discussion

**Figure 1:**
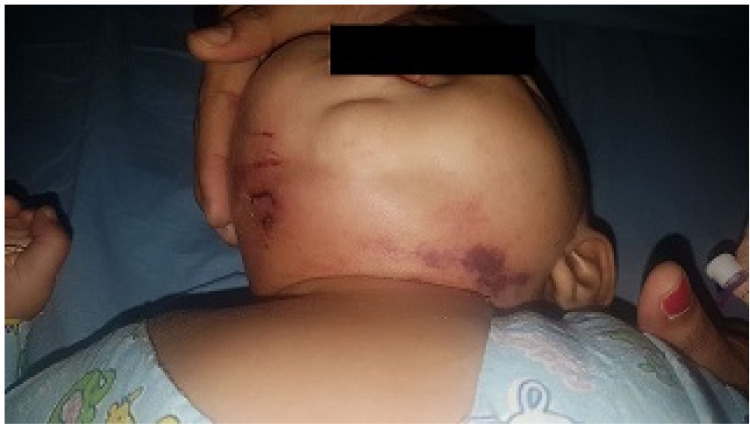
Ludwig's angina presentation in the six-month-old patient.

**Figure 2:**
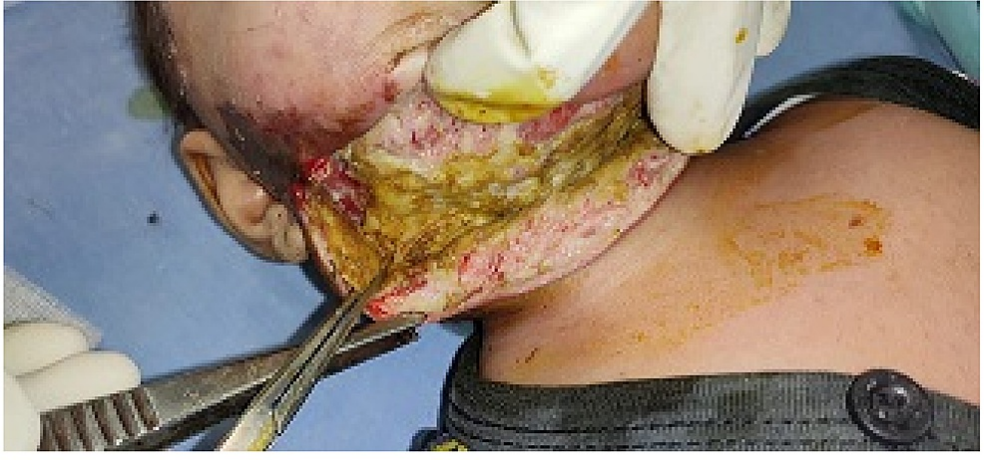
Surgical decompression from one angle of mandible to another.

**Figure 3:**
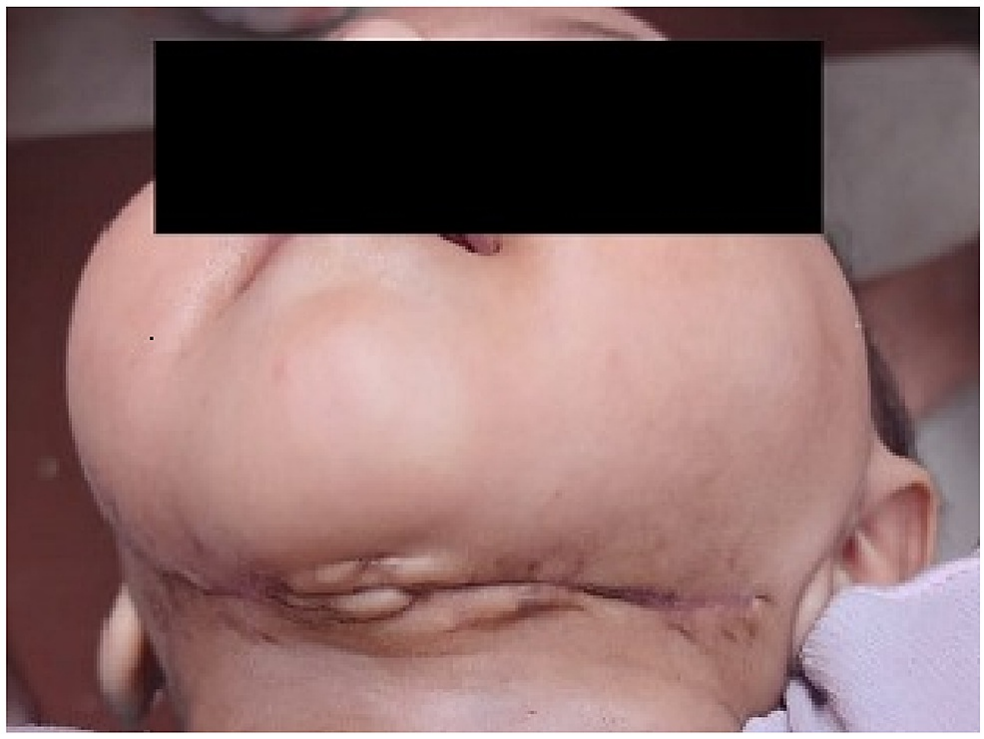
Healed wound post decompression and after secondary suturing.

Ludwig’s angina has been reported as a rare clinical condition and mortality observed in the pre-antibiotic era was 50% [7]. However, with the advent of current antimicrobial therapies and awareness regarding dental health, mortality has reduced to around 10% [8,9]. The male-to-female ratio of treated patients in the present study was 1:1.5, which is different from other studies, in which ratio of male patients was higher. Linas et al. and Fakir et al. showed male-to-female ratio of 1.3:1 and 2.57:1, respectively [10,11]. In this study, the mean age of patients is 38.86 years, which is a little lower than the mean age of 43.18 years found in the study held by Linas et al. [10]. Ludwig’s angina was unusual in children. But in this study, it was found in four children, including one female infant patient (six months old). Photos of clinical presentation, surgical decompression, and healed wound after decompression of this female infant are shown in Figures 1, 2, 3, respectively. Like Fakir et al., in this study, 21 patients (70%) were from lower socioeconomic classes which may be due to poor oral hygiene [11].

The trend of late presentation of patients to hospital was observed in this study. This trend was also reported by Ugboko et al. in Southwest Nigeria [12]. One should expect that patient report to health system early because Ludwig’s angina is a rapidly progressing disease with life-threatening complications. Patients who present early were managed mostly with conservative management and some with surgical intervention with low risk. Late presentation was mostly seen in unemployed, neglected, mentally retarded, old patients, or else, in patients who didn’t come until they developed difficulty in swallowing and/or breathing. In delayed presented patients, even after very aggressive management prognosis was poor.

In this study, dental infection from the mandibular molars was the most common causative factor of Ludwig’s angina (80%), while diabetes mellitus was the second most common associated factor constituting 36.67% of total patients which was like the Fakir et al. [11]. According to Tschiassny, attachments of roots of second and third lower molars extend below the mylohyoid line and close to inner cortex of mandible leading to submaxillary space involvement, while attachment of roots of other lower teeth remains above the line and close to outer cortex of mandible, therefore sublingual space involvement found more in their infections [13]. The cause of cellulitis in the female infant, as shown in Figure 1, was unknown insect bite. Etiology like tonsillar infection, HIV-AIDS, autoimmune conditions, etc. were not observed in this study.

Neck swelling, neck pain, fever, dental problems, and dysphagia were common clinical features observed in this study which was similar to the case series of Fakir et al. [11]. More than one symptom was present in all patients in this study, while according to Ovassapian et al., pain in floor of mouth and anterior neck, dysphagia, odynophagia, and respiratory distress were the most common presenting symptoms [14]. Other common symptoms observed in this study were swelling in floor of mouth, fetid breath, muffled voice, trismus, and respiratory distress. In all patients, on palpation diffuse, firm, non-pitting, indurated, tender swelling was found in submental and bilateral submaxillary space in supraomohyoid neck which may extend further in the lower neck in some cases.

Treatment protocol in various past studies and the current study was almost the same. Prasad et al. described four principles of management - (1) sufficient airway management, (2) early and aggressive injectable broad-spectrum antibiotics, (3) incision and drainage and removal of primary cause if found any (i.e., odontogenic cause), (4) adequate hydration and nutrition support [15].

Initial empirical antimicrobial therapy targeted against Gram-positive organisms and oral anaerobes. Intravenous amoxicillin+clavulinic acid or cefoperazone+sulbactam, with intravenous metronidazole/clindamycin and aminoglycosides like gentamicin/amikacin were given empirically to all patients initially in this study, followed by antibiotics as per culture and sensitivity report. In some previous studies, intravenous steroids were used to relieve soft tissue swelling and edema [16]. Systemic corticosteroid was also used in eight patients in this study.

Polymicrobial type of flora is commonly seen in Ludwig’s angina. Streptococcus, Staphylococcus, and Bacteroides species are the most commonly found organisms followed by some Gram-negative organisms like Klebsiella, *Haemophilus influenzae*, *Pseudomonas aeruginosa*, and Proteus species [17]. Gram-positive organisms like Streptococcus and Staphylococcus and Gram-negative organisms like *Escherichia coli* were commonly isolated from pus culture in this study which is similar to the case series of Fakir et al. [11].

Diagnosis in Ludwig’s is mostly based on clinical findings though the contrast-enhanced CT scan is more helpful in the prediction of drainable pus and extension of spread in deep neck abscesses [18]. Most of the patients in this study were from lower and middle socioeconomic class and were non-affording for CT scans, so CT scan was done only in six patients. Instead of CT scan, ultrasonography and plain soft tissue x-ray were done for most of the cases, which are more cost-effective and gave information regarding site and size of the collection, extent of spread, and site of pus collection where maximum fluctuation was palpable. Only clinical findings have low efficacy in the prediction of site of drainage while combined with CT scan finding, the accuracy is 89% and sensitivity and specificity is 95% and 89%, respectively [19].

Airway monitoring and protection have been identified as the most important aspect of the management of Ludwig’s angina [20,21]. All patients were managed in the intensive care unit. Our management of the airway included placement of patients in cardiac position with the use of oropharyngeal airway and tracheostomy under local anesthesia, whenever indicated. Endotracheal intubation with flexible fiberoptic bronchoscopy is another method used to secure airway. In this study, six patients (20%) required tracheostomy, and this rate is almost double than the case series in Fakir et al. [11]. Many patients with co-morbidities presented late with diffuse swelling, and at the time of surgical decompression, they required tracheostomy.

The indications of surgical decompression are clinically palpable fluctuation or crepitation of gas, serous or pus collection in ultrasonography, radiological evidence of air in soft tissue, and no clinical improvement in 48 hours after starting antimicrobial therapy [22]. Out of total 30 patients, 24 (80%) required surgical decompression in this study, which was similar to the case series of Fakir et al. [11]. Timely surgical decompression reduces complications and thus mortality also.

Sepsis, pneumonia, asphyxia, empyema, pericarditis, mediastinitis, and pneumothorax are possible complications of Ludwig’s angina [14]. The most common complication reported in this study were necrotizing fasciitis (16.66%) followed by septicemia (10%) and death (10%). While according to the case series of Fakir et al., the most common complications are necrotizing fasciitis (8%) and septicemia (8%) followed by mediastinitis (6%) [11]. Many case series reflect mortality in Ludwig’s angina resulting from mediastinitis, severe sepsis, and laryngeal spasm in past [8,22]. In the current study, septicemia found the most common complication which leads to mortality followed by DIC and/or cardiac arrest. Mediastinitis-related mortality was also observed in one pregnant female in this study. This study has some major limitations. It is a single-center, retrospective study with limited cases. In future, it can be done on multi-centric basis with more cases of Ludwig's angina.

## Conclusions

Despite improved outcomes with higher antibiotics and available advanced surgical facilities, Ludwig's angina still remains a potentially life-threatening disease in ENT. Dental caries, uncontrolled diabetes mellitus, and malnutrition are commonly associated conditions. With early diagnosis, close airway observation, aggressive intravenous antimicrobial treatment, and timely surgical intervention, one can reduce morbidity and mortality.
